# Rare Presentation of a Rare Disease: Signet-Ring Cell Gastric Adenocarcinoma in Rothmund-Thomson Syndrome

**DOI:** 10.7759/cureus.11865

**Published:** 2020-12-03

**Authors:** Zeeshan Ali, Pritika S Manaktala, Saro Sarkisian, Muhammad Rizvi

**Affiliations:** 1 Hematology and Oncology, Lehigh Valley Cancer Institute, Allentown, USA; 2 Internal Medicine, Canton Medical Education Foundation, Canton, USA

**Keywords:** recql4 gene, gastric adenocarcinoma, rothmund-thomson syndrome, poikiloderma

## Abstract

Rothmund-Thomson syndrome (RTS) is an exceedingly infrequent genetic disorder characterized by a multitude of skin findings collectively known as poikiloderma. In normal cells, the RECQL4 gene is involved in DNA replication and repair. RTS is caused by a mutation in the RECQL4 gene, which results in increased predilection to develop various malignancies. Osteosarcomas and skin cancers are typically associated with this syndrome. We present a rare case of signet-ring cell gastric adenocarcinoma in a patient with RTS.

## Introduction

Rothmund-Thomson syndrome (RTS) is a rare autosomal recessive genodermatosis that presents in infancy. Clinical features include short stature, sparse scalp hair, sparse or absent eyelashes, eyebrows, skeletal abnormalities, juvenile cataracts, radial ray defects, premature aging, and a constellation of classical skin findings, known as poikiloderma. RTS is associated with a predisposition to developing certain cancers, particularly osteosarcoma and skin cancer [1]. To our knowledge, there has been only one published case report of gastric malignancy in association with this congenital disorder [2]. We describe a case of signet-ring cell gastric adenocarcinoma in a patient with RTS.

## Case presentation

An 18-year-old female patient with a history of RTS and deafness presented to us with abdominal pain, which had been progressively worsening over the preceding one year. It was associated with nausea, vomiting, early satiety, and progressive weight loss due to poor oral intake. Previously, her abdominal pain was thought to be caused by gastroesophageal reflux disease. However, a trial of proton pump inhibitor therapy had not improved her symptoms. On physical examination, she was noted to be cachectic. Findings consistent with the diagnosis of RTS included fish-like facies, congenital deafness, widely distributed poikiloderma (areas of hyper and hypopigmented areas giving the appearance of mottling), and telangiectasias. She had moderate abdominal tenderness.

**Figure 1:**
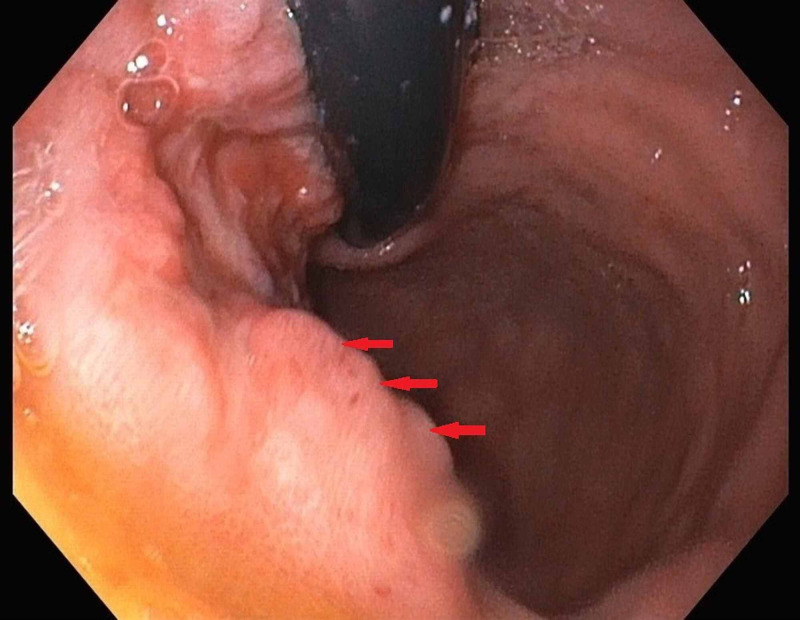
Arrows indicate the irregular areas of gastric mucosa as seen on esophagogastroduodenoscopy

**Figure 2:**
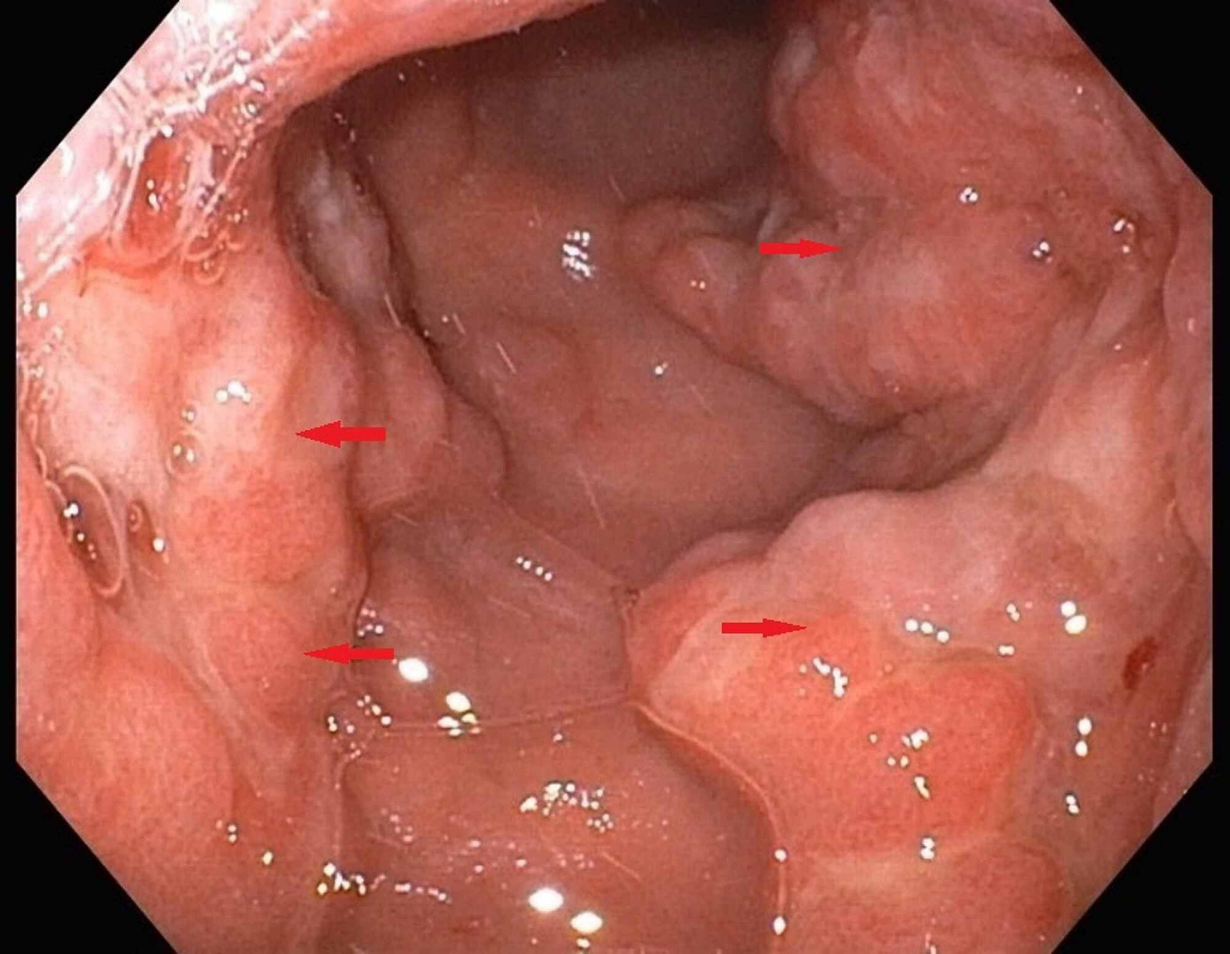
Arrows indicate the irregular areas of gastric mucosa as seen on esophagogastroduodenoscopy

**Figure 3:**
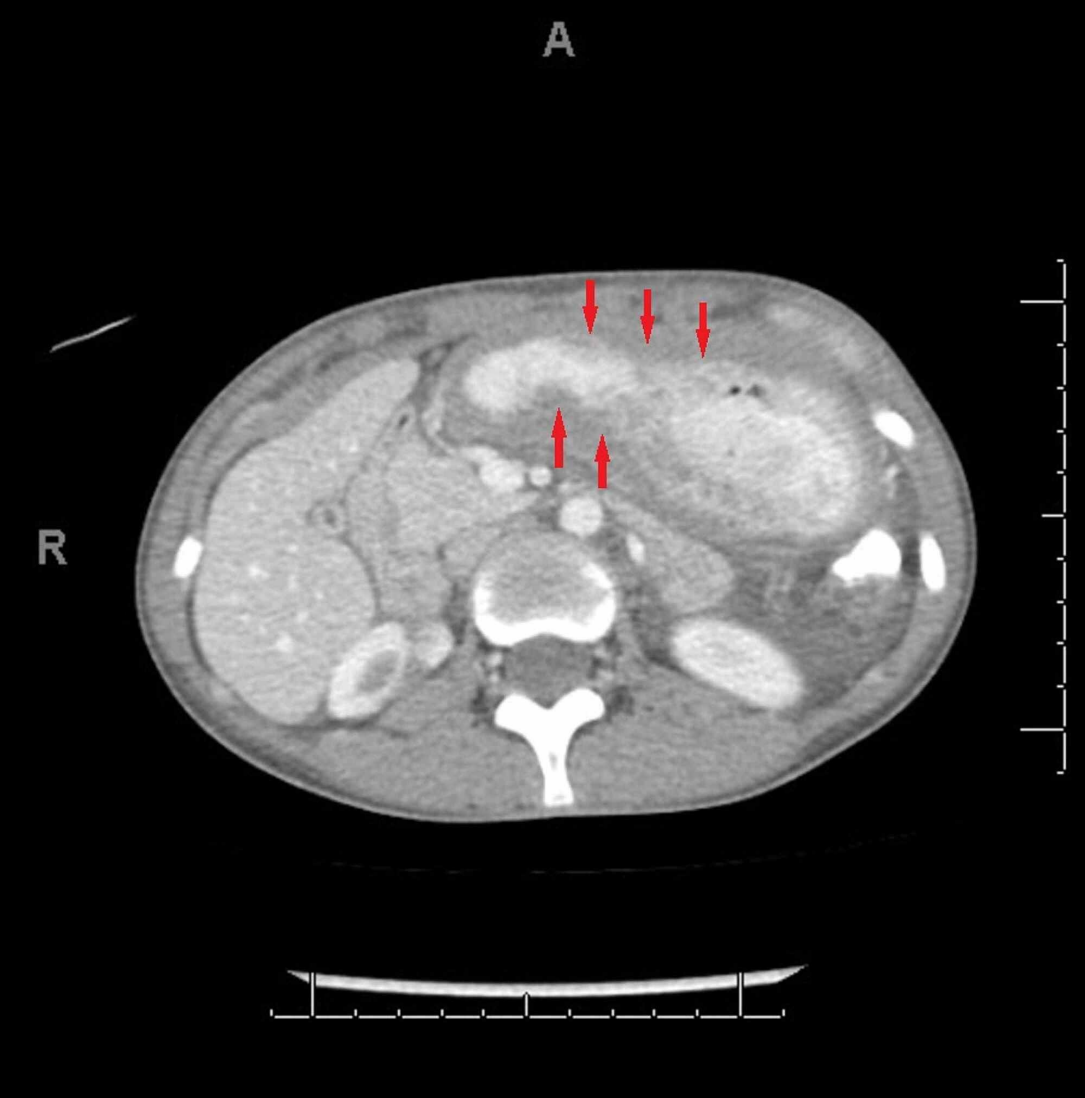
CT scan axial view: arrows indicate extensive infiltration of the stomach wall with the malignant process

**Figure 4:**
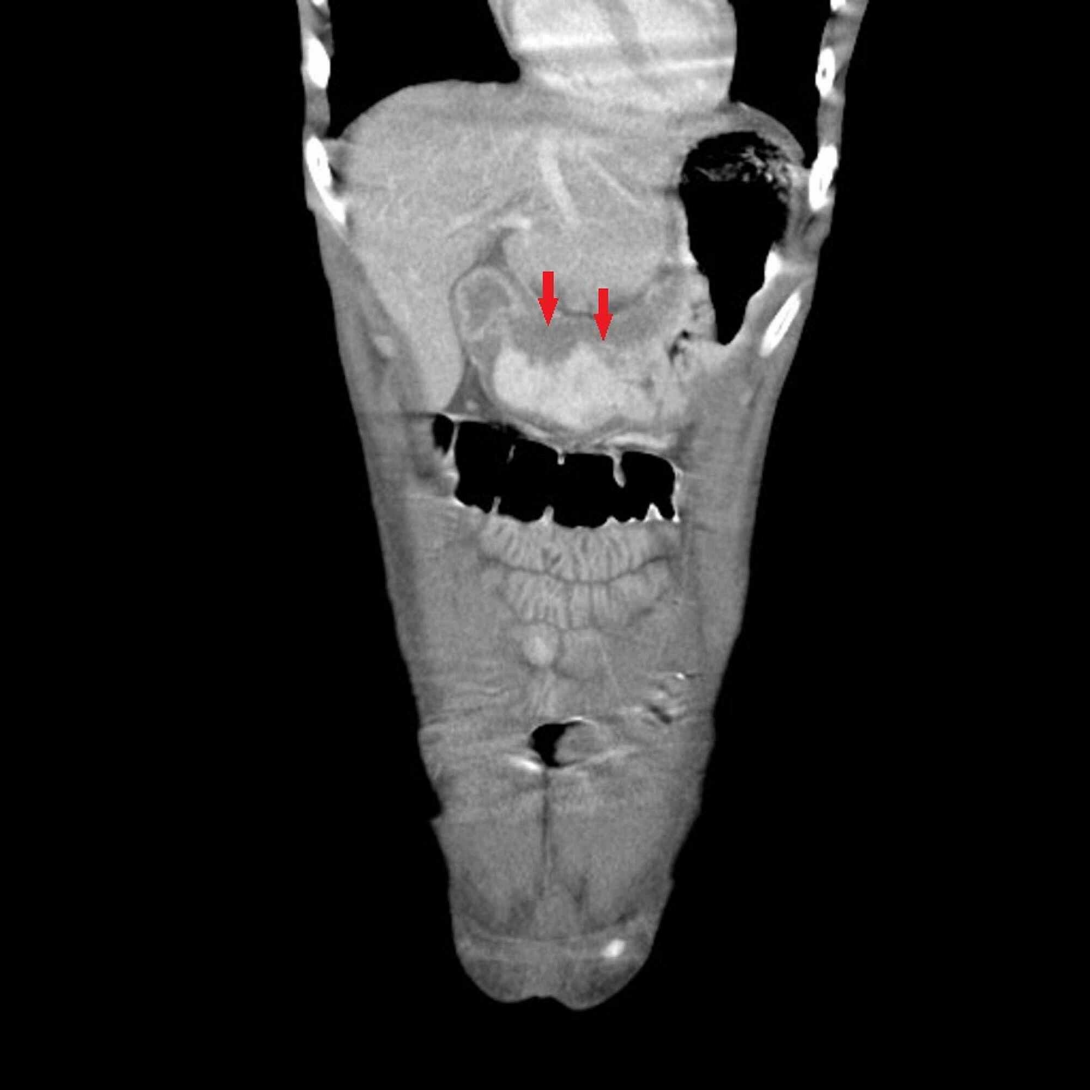
CT scan coronal view: arrows indicate extensive infiltration of the stomach wall with the malignant process

Contrast-enhanced CT scan of the abdomen and pelvis showed gastric and colonic wall thickening, small volume ascites, and borderline upper abdominal and retroperitoneal lymphadenopathy. Esophagogastroduodenoscopy (EGD) with endoscopic ultrasound EUS revealed significantly abnormal mucosa of the gastric cardia. Findings included variegation in mucosal appearance, ulceration, and thickening, highly suggestive of a diffuse infiltrative process with circumferential extension to the level of the pylorus. Figures 1-2 reveal the irregular areas of gastric mucosa as observed on EGD. Extensive infiltration of the stomach wall can be seen on axial (Figure 3) and coronal (Figure 4) CT images.

Gastric biopsies confirmed poorly differentiated adenocarcinoma with signet-ring cell features. Staging laparoscopy showed no peritoneal involvement. She was provided enteral nutrition via jejunostomy tube in preparation for the surgery. Subsequently, she underwent a total gastrectomy with lymph node dissection. R1 resection was achieved with positive proximal and distal surgical margins. Lymphovascular invasion and lymph node involvement (seven out of 16 resected lymph nodes were positive for malignant cells) were observed in the surgical specimen. Pathologic stage was finalized as IIIB (pT3N3aM0) with histologic grade G3 (poorly differentiated).

Because of poor baseline performance status and severe protein-calorie malnutrition, she was deemed unfit for adjuvant chemotherapy. After a detailed discussion with her family, it was decided to closely monitor the patient with serial body imaging. Unfortunately, her postoperative course was complicated by recurrent episodes of large bowel obstruction, eventually requiring surgical bypass of the obstruction by colo-colostomy (side to side anastomosis of the cecum to the sigmoid colon). Intraoperatively, there was evidence of peritoneal recurrence of gastric cancer, which was confirmed on biopsy of one of the serosal lesions. Her postoperative course was complicated by the development of bilateral pelvic abscesses and septic shock. She underwent CT-guided insertion of the pelvic drainage catheters. Despite our best efforts to treat the infection, her clinical condition continued to worsen. Ultimately, she was transitioned to the inpatient hospice unit.

## Discussion

Rothmund-Thomson syndrome (RTS) is a very rare genetic disorder. To date, only 300 cases have been reported in the medical literature [1]. It is transmitted in an autosomal recessive pattern, and in the vast majority of cases, it is caused by mutations in the RECQL4 gene located at chromosome 8q24, which codes for a protein family called RECQL4 DNA helicases. These DNA helicases are involved in the replication and repair of DNA [3]. RECQL4 mutations can lead to the production of an abnormally short, nonfunctional version of the RECQL4 protein or prevent cells from making any of this protein at all. A defective or deficient RECQL4 protein prevents normal DNA replication and repair, resulting in a high predilection for developing malignancies.

RTS was first described by the German ophthalmologist, Rothmund in 1868 and later in 1936 by an English dermatologist, Thomson [3]. Typically, dermatologic abnormalities manifest between three and six months of age. Patients develop erythema, swelling, blistering, telangiectasia, and punctate atrophy of the skin (collectively, these findings are known as poikiloderma, which is the hallmark of RTS). Small stature, skeletal and dental abnormalities, juvenile cataracts, sparse hair, eyelashes, eyebrows have also been reported [1, 4]. Hyperkeratotic lesions are noted in about one-third of the patients, and sensorineural deafness has also been described [1].

An extensive literature search revealed only one case of gastric carcinoma in a patient with RTS, which was reported in 1975 [2]. One other report described a case of invasive mucinous adenocarcinoma involving the second part of the duodenum [3]. Patients with RTS have a known predisposition to various malignancies due to the defect in DNA replication and repair, osteosarcoma being the most common with a mean age of onset around 14 years. Skin involvement in the form of squamous cell carcinoma, basal cell carcinoma, and Bowen’s disease has also been reported; however, the mean age of onset is about 34 years. Osteosarcoma has an estimated prevalence of 30% compared to skin cancers, which have an estimated prevalence of about 5% [3]. Non-malignant gastrointestinal system involvement can manifest as esophageal or pyloric stenosis, anal atresia, annular pancreas, and rectovaginal fistula. Feeding problems, chronic emesis, and diarrhea can be seen in infancy, but they usually resolve in later childhood [1]. Gastric cancer is rare in this patient population, and our patient was only the second known case of gastric malignancy associated with RTS. Due to the rarity of this cancer in this age group and in this exceedingly rare genetic disorder, several months had elapsed prior to the initiation of extensive workup, which led to her cancer diagnosis. Unfortunately, her performance status had declined significantly by that time, making her a poor candidate for adjuvant chemotherapy.

## Conclusions

Owing to the rarity of Rothmund-Thomson syndrome (RTS), routine cancer screening may not be feasible in patients suffering from this congenital disorder. However, our case highlights the importance of recognizing the association of RTS with various malignancies to promptly initiate diagnostic workup based on pertinent clinical presentation. Such an approach may help improve outcomes for this patient population.

## References

[1] Larizza L, Roversi G, Volpi L (2010). Rothmund-Thomson syndrome. Orphanet J Rare Dis.

[2] Diem E (1975). The Rothmund-Thomson-syndrome. A case report. Hautarzt.

[3] Nadeau K, Brule M (2018). Gastrointestinal malignancy presenting with a Virchow's node in a patient with Rothmund-Thomson syndrome. Case Rep Genet.

[4] Wang LL, Plon SE (1999). Rothmund-Thomson Syndrome. https://www.ncbi.nlm.nih.gov/books/NBK1237/.

